# Winter Exercise and Speleotherapy for Allergy and Asthma: A Randomized Controlled Clinical Trial

**DOI:** 10.3390/jcm9103311

**Published:** 2020-10-15

**Authors:** Johanna Freidl, Daniela Huber, Herbert Braunschmid, Carina Romodow, Christina Pichler, Renate Weisböck-Erdheim, Michaela Mayr, Arnulf Hartl

**Affiliations:** Institute of Ecomedicine, Paracelsus Medical University Salzburg, 5020 Salzburg, Austria; johanna.freidl@pmu.ac.at (J.F.); daniela.huber@pmu.ac.at (D.H.); Herbert.Braunschmid@stud.sbg.ac.at (H.B.); Carina.Romodow@traunstein.bayern (C.R.); christina.pichler@pmu.ac.at (C.P.); renate.erdheim@pmu.ac.at (R.W.-E.); michaela.mayr@pmu.ac.at (M.M.)

**Keywords:** winter exercise, speleotherapy, exhaled nitric oxide, allergy, asthma, allergic rhinitis, united airway disease

## Abstract

(1) Background: The prevalence of allergic respiratory diseases is still rising and efforts towards holistic treatments should be made. Although speleotherapy is widely applied in Europe to treat chronic airway diseases, the existing scientific evidence is rather low. Recreational winter exercise has been shown to improve allergic airway inflammation, but little is known about the combined effects of speleotherapy and recreational winter exercise. (2) Methods: In this clinical study we investigated the effects of winter exercise and speleotherapy on adults with allergic rhinitis and/or asthma. The speleotherapy group (n = 23) participated in a ten-day combined winter exercise and speleotherapy program and the exercise group (n = 18) joined a full-day winter sports program. The effects on allergic airway inflammation, quality of life, spirometry and cardiorespiratory fitness were assessed. (3) Results: No significant effects were found for fractional exhaled nitric oxide or nasal nitric oxide. Quality of life (*p* < 0.001 time effect) and allergic symptoms (*p* < 0.001 time effect) were improved in the speleotherapy and in the exercise group. (4) Conclusions: Winter exercise alone and winter exercise in combination with speleotherapy improve quality of life and allergic symptoms in adults with allergic rhinitis and/or asthma. Further studies are required to investigate the specific effects of speleotherapy. To our knowledge, this is the first investigation examining speleotherapy in combination with winter exercise. Recreational outdoor winter exercise and speleotherapy may be recommended for highly functioning patients with good disease control.

## 1. Introduction

Respiratory allergies affect 20–30% of the European population and the prevalence of these diseases is still on the rise. By 2025, every second European will suffer from at least one allergic disease, independent of age, social or geographical distinction [1,2]. Facing this progression, allergic diseases must be recognized as growing public health issue and the development of population-based cost-effective and side-effect free therapies is more relevant than ever [1]. Traditionally, allergic rhinitis and asthma have been treated as two distinct diseases, but with the emerging concept of the united airway disease, a more integrated view of respiratory allergies has been developed [2,3,4]. The standard therapy of allergic respiratory disease is mainly focused on pharmacological interventions, allergen avoidance or specific immunotherapy [5,6]. Beside these approaches, regular physical activities or sojourns at high altitude have been shown to improve respiratory symptoms, quality of life and exercise capacity [7,8]. Furthermore, recreational outdoor winter exercise improves allergic airway inflammation, measured as fractional exhaled nitric oxide, and allergic symptoms, as our working group could recently show [9].

**Figure 1:**
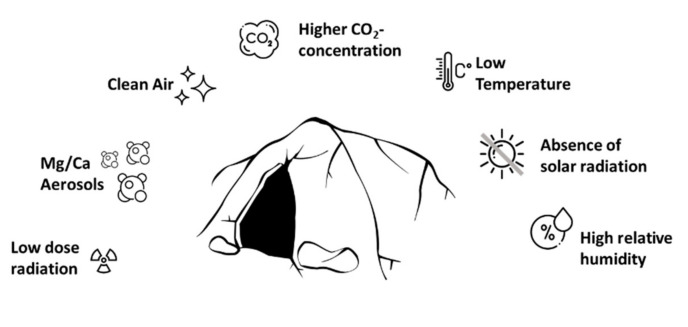
Complexity of speleotherapy. Mines and caves are characterized by a specific microclimate.

Another complementary therapy option for chronic respiratory conditions is speleotherapy. Speleotherapy (from the Greek “speleon”—cave) is a special kind of inhalation therapy, which uses the specific microclimate of mines and caves to treat chronic airway diseases. This specific microclimate, also called speleoclimate, is mainly characterized by stable temperature, slow air motion, absence of pollutants and allergens, special physicochemical properties like high relative humidity, higher CO_2_ levels, low dose radiation and deprivation of external stimuli [10,11,12]. Depending on the specific speleoclimate three main types of speleotherapy locations can be distinguished: high temperature and high rate of air ionization (e.g., Gastein Healing Gallery, Austria), low temperature and high rates of air ionization, and low temperature caves with low rates of air ionization (e.g., Bad Bleiberg “Friedrichstollen”, Austria) [13]. Scientific research on speleotherapy started in the 20th century resulting in the approvement of speleotherapy as therapy option for respiratory diseases as well as for certain skin diseases [14,15,16]. Although speleotherapy is widely known in Central and Eastern Europe and many active speleotherapy locations are currently offering therapies [17,18,19,20], the existing scientific evidence is rather low or simply not accessible, as most literature from Eastern Europe is not available in English [14,21]. There is an extensive geographical distribution of speleotherapy facilities in Eastern Europe, but outside the Eurasian continent speleotherapy is actually unknown in scientific literature [22]. However, the specific curative factors of speleotherapy are still not fully understood. Among the complex and highly individual physicochemical features of speleotherapy-locations (see Figure 1), several potential curative factors can be identified:

### 1.1. High Relative Humidity

The relative humidity in caves with low temperature is about 93–98% [23,24]. With the temperature increase during inhalations, the relative humidity inside the airways drops from 93–98% to about 30–33%. From this temperature-humidity dependency, early research concluded that the lowering of humidity within the airways leads to a water drain from the surrounding tissue into the bronchial mucus, thus diluting the mucus and increasing muco-ciliary clearing rate [23]. Mucus clearance is an essential innate immune feature of the airways. Therefore, efficient mucus clearance is needed for respiratory health [25].

### 1.2. Cave Aerosols

In general, an aerosol is defined as a suspension of small liquid or solid particles dispersed in a gaseous medium. Cave aerosols contain high amounts of dissolved substances like magnesium or calcium [11,23,26]. Magnesium is known for its spasmolytic effect on smooth muscle cells. Inhalation of magnesium sulfate leads to a faster improvement of moderate to severe asthma attacks in human and animal models [27,28]. Also, relatively high amounts of calcium particles in the cave atmosphere may be capable of inducing anti-inflammatory effects. Lung deposition models simulating the inhalation of Ca^2+^-cave aerosols by Alföldy and colleagues show that 39% of the aerosols were deposited between the 6th and 15th airway generation, which is the region mostly affected by asthma [26].

### 1.3. Slow Air Motion and Air Pollution

Air pollution poses a health risk and is associated with exacerbations of allergic respiratory disease [29]. Caves are characterized by very clean air and low fine dust concentrations, even when they are surrounded by a highly polluted environment [30]. Cave ventilation is controlled by external temperatures. However, cave ventilation and morphology strongly influence how aerosols and particles are transported and deposited. Coarse aerosols are mostly deposited in the entrance zone. Pollen are also most abundant close to the entry as the cave air flow is too weak to transport such large aerosols further inside [11,31,32].

### 1.4. Radiation and Ionization

Radon is a radioactive noble gas which often accumulates in underground areas. For caves, high concentrations of ^222^ Rn ranging from 0.1 to 20 kBq/m^3^ have been reported. The radon concentration underlies great seasonal fluctuations and is categorized as harmless [33,34,35]. The concept of radiation hormesis proposes that low doses of ionizing radiation induce beneficial biological effects [36,37].

### 1.5. Absence of Solar Radiation and Ozone

Although, ozone has a protective role against ultraviolet radiation, high concentrations at ground level are harmful and negatively affect the respiratory and cardiovascular system [38]. In the subterranean environment of caves and mines sun light and solar radiation are not present and hence no ozone is present [39].

Taken together, the curative effects of speleotherapy may be based on the interference of clean air, mucolytic-, spasmolytic- and anti-inflammatory effects. However, the speleoclimate is a highly complex interaction-network, which is dependent on the specific cave situation [11]. Although the curative factors are relatively unknown, some clinical studies have investigated the effects of speleotherapy. Beamon et al. screened, in a Cochrane Review, all available speleotherapy studies before 2001. Three trials (total n = 124, patients with asthma bronchiale) were included, but only one study fully met the inclusion criteria [21]. Work by Novotny showed improvements in lung function in children after a three weeks treatment in the Gastein Healing Gallery (high temperature and high radiation) [13]. A controlled randomized multicenter study by Gaus et al. in 2010 also showed a significant improvement in children after a three weeks treatment period in low temperature caves in Germany [40]. Despite the fact, that the scientific evidence for speleotherapy is rather low, speleotherapy could be a potential alternative treatment option for respiratory diseases [41,42,43].

Previous findings from the WESPAA-Study (Winter exercise and speleotherapy for allergy and asthma, ISRCTN88277657) indicate beneficial effects of recreational winter exercise alone on allergic airway inflammation [9]. This work focuses on the specific effects of recreational winter exercise in combination with speleotherapy.

## 2. Materials and Methods

### 2.1. Study Design and Settings

**Figure 2:**
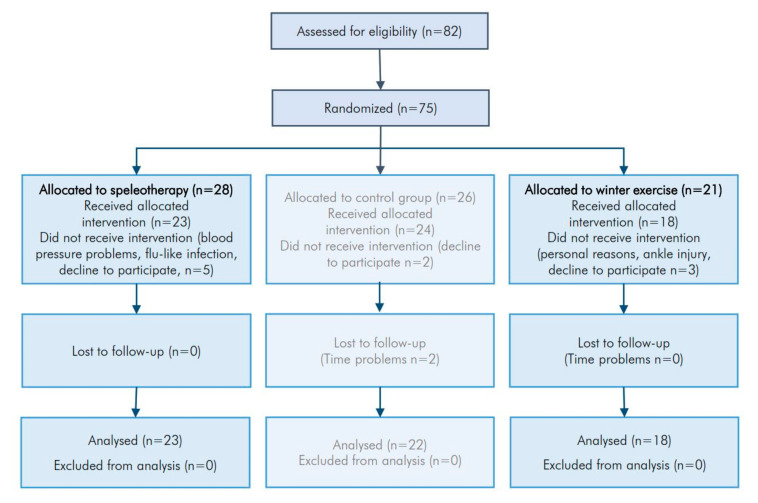
Study flowchart of included and excluded patients.

The presented data is part of the WESPAA-Study (ISRCTN88277657) which investigates the effects of winter exercise and speleotherapy on patients suffering from allergic rhinitis and/or allergic asthma. Two intervention groups, aspeleotherapy group (Table S1), an exercise group (Table S2) and one control group (Table S3) were included. This work is focused on the comparison of winter exercise alone versus winter exercise in combination with speleotherapy, thus completing the already published results from the analysis of the control and the exercise group [9]. The allocation ratio was set at equal sample size for all groups. The study protocol was approved by the Ethics Committee of Salzburg (415-E/1553/2-2012). Study flowchart is presented in Figure 2.

### 2.2. Participants

Participants were recruited via newspaper and online advertising between November 2012 and February 2013. The following inclusion criteria were applied: age 18–55 years, house dust mite sensitization (RAST > 1; positive PRICK-Test or total IgE > 0.7 kU/l), controlled allergic rhinitis and/or allergic asthma, physical ability to perform recreational winter exercise (including moderate skiing skills) (see Table 1). The following exclusion criteria were applied: uncontrolled asthma (Asthma Control Test [44] <20), exercise induced bronchoconstriction, lung function disorder, acute infection or fever, malignant neoplastic disorders, orthopedic diseases, cardiovascular diseases, uncontrolled metabolic diseases and pregnancy. Prior to enrollment, an informed consent was obtained from all participants.

### 2.3. Intervention

Both intervention groups spent a ten-day holiday in the Hohe Tauern National Park (Salzburg, Austria) and were hosted in the same certified allergy-friendly hotels (Hohe Tauern Health, www.hohe-tauern-health.at). These hotels meet evidence-based quality criteria for the clientele of allergic and asthmatic guests including, e.g., maximum permitted concentrations of major indoor allergens. To refresh basic knowledge about asthma self-management and safety aspects, both groups received a one-hour asthma class with a physiotherapist. In March 2013, the speleotherapy group participated in eight speleotherapy sessions in the former copper mine “Schaubergwerk Hochfeld” (47°13’19.8” N 12°16’26.4” E) and in moderate winter exercise activities (see Table 1). The “Schaubergwerk Hochfeld” was established in 1773 and was reactivated as a show mine between 1990 and 1992. To reach the exposure site, participants had to walk through a 400 m long rock tunnel inside the mountain. During speleotherapy, all participants were seated in deck chairs. Each speleotherapy session lasted 1.5 h. Afterwards the speleotherapy group participated in a winter sports program. In December 2013, the exercise group completed a daily winter sports program without speleotherapy (see Table 2).

### 2.4. Data Collection and Outcomes

**Figure 3:**
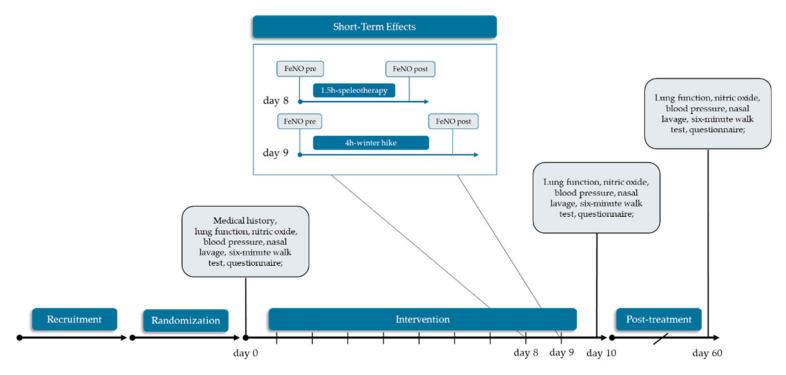
Study schedule. This work is focused on speleotherapy and winter exercise solely. No data from the control group are shown.

Fractional exhaled nitric oxide (FeNO) and the German version of the RhinAsthma Quality of Life Scale [45] were defined as primary outcomes. The RhinAsthma Quality of Life Scale measures the impairment of quality of life in patients with rhinitis and asthma. The following parameters were selected as secondary outcomes: spirometry, nasal NO, six-minute walk test (6MWT), differential blood count, eosinophilic cell count from nasal lavage, muco-ciliary clearance time and inverse visual analogue scale (VAS) regarding general health status and allergy symptoms. Data were anonymized by four-digit-ID. Medical examinations at baseline (T0; day 0) and after the intervention (T1; day 10) were performed in a mobile lab in Mittersill, Austria. Follow-p examinations (T2; day 60) were completed at the Paracelsus Medical University in Salzburg, Austria. Forearm venous blood (BD Vacutainer ^®^ system—Becton Dickinson AG, Vienna, Austria) was collected at all time points by trained medical staff and differential blood count was performed by the Institute for Medical and Chemical Laboratory Diagnostics at the Paracelsus Medical University Salzburg, Austria. To assess short-term effects of a single speleotherapy-session and a single winter hike, additional FeNO-measurements were performed at day 8 in the speleotherapy group and at day 9 in the exercise group. No specific lifestyle recommendations were given for the non-treatment time in any group. Study schedule is presented in Figure 3.

#### 2.4.1. Physical Characterization of the Speleotherapy Location

Fine dust concentrations, temperature and relative humidity in the mine were measured with an Aerosol Spectrometer model 1.108 (GRIMM Aerosol Technik Ainring GmbH & Co. KG, Ainring, Germany). Time interval for measurements was set at 6 s. The radon concentration was measured with a Radon/Thoron-Monitor 1688 (SARAD GmbH, Dresden, Germany) and CO_2_ concentration was measured with CM100 CO_2_ meter (VOLTCRAFT Conrad Electronic AG, Wollerau, Switzerland).

#### 2.4.2. Fractional Exhaled Nitric Oxide and Nasal Nitric Oxide

As a measure of allergic airway inflammation, fractional exhaled nitric oxide (FeNO) and nasal nitric oxide (NO) was assessed by NioxMino ^®^ (Aerocrine AB, Solna, Sweden) according to the ATS/ERS guidelines (American Thoracic Society/European Respiratory Society Statement) [46]. Data from 9 participants from the exercise group were lost at baseline due to a technical fault of the NioxMino ^®^-device. Only participants with a complete series of FeNO and nasal NO measurements were included in the statistical analysis (n = 9). On day 8 (T0.8) short-term effects of one single speleotherapy session were assessed by two additional FeNO-measurements before and after speleotherapy. Short-term effects of winter exercise were evaluated at day 9 (T0.9) by additional FeNO-measurements before and after a 4-h winter hike (see Figure 2).

#### 2.4.3. Spirometry and Six-Minute Walk Test

Forced expiratory maneuver was performed according to the manufacturerߣs-(EasyOne, ndd Medical Technologies, Zurich, Switzerland) and ATS/ERS guidelines [47]. The following parameters were analyzed: forced expiratory volume (FVC) (%), forced expiratory volume in 1 s of forced vital capacity (FEV_1_) (%), FEV_1_/FVC (%), peak expiratory flow (PEF) (%) and mean forced expiratory flow between the 25% and 75% of the FVC (MEF_25–75%_). Six-minute walk test (6MWT) was performed according to manufacturer’s- (SpiroPalm, COSMED srl, Rome, Italy) and ATS guidelines [48]. Following parameters were analyzed: distance walked in 6 min (%), BORG-Scale,) peak ventilation frequency (1/min) and peak minute ventilation (L/min). Reference values for the predicted six minute walk test distance are described by Chetta et al. [49].

#### 2.4.4. Nasal Lavage and Saccharin Test

Cell counts from nasal lavage were obtained from every participant as described in our previous work [9]. For the assessment of the muco-ciliary clearance time, a saccharin particle was placed in the inferior turbinate of the nasal cavity [50].

### 2.5. Statistcial Analysis

The statistical analyses were carried out with the R-GNU software environment (General Public License, R Foundation for Statistical Computing, Vienna, Austria, version 3.4.4). All variables are expressed as mean ± standard deviation and missing data were replaced by the LOCF-method (last outcome carried forward). The statistical significance level was set at α = 0.05 and multiple testing was corrected according to Holm-Bonferroni. Fully nonparametric variance-type tests form the nparLD package [51] were applied for longitudinal data analysis. Within an F1-LD-F1 model, treatment (speleotherapy vs. exercise) was included as whole-plot-factor and time (T0, T1, T2) was defined as sub-plot-factor. Effects for treatment, time and the interaction between treatment and time were evaluated. In case of a significant main effect for time, further F1-LD-F1 models were applied for single time point comparisons. For the analysis of short-term effects of FeNO a second F1-LD-F1-model with time and treatment was applied. Due to the small sample size, only results from the ATS-type tests are presented.

Relative treatment effects (RTE) were used as a measure of effect. The RTE can take a range of values between 0 and 1. A value of 0.5 indicates that there is no effect. An RTE > 0.5 in the exercise group means a tendency for subjects in the exercise group to score at least as high (or higher) as a randomly chosen subject from the entire dataset. On the other side, an RTE of 0.25 in the exercise group means that the probability of a randomly chosen subject from the entire subset having a lower score than a randomly chosen subject from the exercise group is estimated to be 25%.

### 2.6. Randomization and Sample Size

The random allocation of the participants to the study groups was conducted with an open-source add-in (Daniel’s XL Toolbox, 2012; Kullback-Leibler divergence) for the Microsoft Excel ^®^ spreadsheet [52]. No a priori sample size analysis was performed, but the results from the primary outcome FeNO were used to perform a sample size estimation based on unpoled bootstrap simulation with equal group size [53]. Within the bootstrap simulation, F1-LD-F1 models from the nparLD package and ANOVA were applied. The initial seed for the variate generator was set at 1 and the re-sampling process was fixed to 1000 repetitions for each sample size. The percentage of significant results was used as an estimator of power. The sample size simulation was performed for both, the comparison of the exercise and speleotherapy group and for the comparison of the exercise and control group.

## 3. Results

### 3.1. Study Participants and Baseline Characteristics

Out of 75 eligible persons, 28 persons were enrolled for the speleotherapy group in March 2013 and 21 persons were invited for the winter exercise program in December 2013. Five persons from the speleotherapy group declined to participate and two participants were lost during the intervention because of blood pressure problems and a flu-like infection. Within the exercise group, one person declined to participate, and two participants were lost during the intervention due to personal reasons and an ankle strain. The intervention was well tolerated by all participants. No adverse effects were observed.

A total of 23 persons from the speleotherapy group and 18 persons from the exercise group were included in the statistical analysis. Baseline characteristics show significant differences for age and the six-minute walk test distance. The speleotherapy group was younger than the exercise group (t_(32.2)_ = 2.22, *p* = 0.03). Furthermore, the speleotherapy group was characterized by a better six- minute walk test performance (t_(37.1)_ = −2.26, *p* = 0.03).

### 3.2. Environmental Parameters

Ambient air temperature for the exercise program ranged between −5.6 °C to 8.7 °C (air temperature 2 m above ground (ZAMG, Austrian Central Institute for Meteorology and Geodynamics). The former copper mine “Schaubergwerk Hochfeld” is characterized by a constant low temperature (7.98 ± 0.09 °C), high relative humidity (94.91 ± 1.67%) and very low fine dust concentrations (PM 10: 4.40 ± 2.29 μg/m^3^, PM 2.5: 0.58 ± 0.31 μg/m^3^, PM 1: 0.23 ± 0.15 μg/m^3^). Common limits for fine dust concentrations in speleotherapy facilities are set at < 8.5 μg/m^3^ for particles greater than 2.5 μm and < 6 μg/m^3^ for particles smaller than 2.5 μm [54]. Slightly higher CO_2_ concentration (0.055 ± 0.002%) and radon levels of 954.32 ± 123.12 Bq/m^3^ were detected in the mine.

### 3.3. Fractional Exhaled Nitric Oxide, Nasal Nitric Oxide and Spirometry

**Figure 4:**
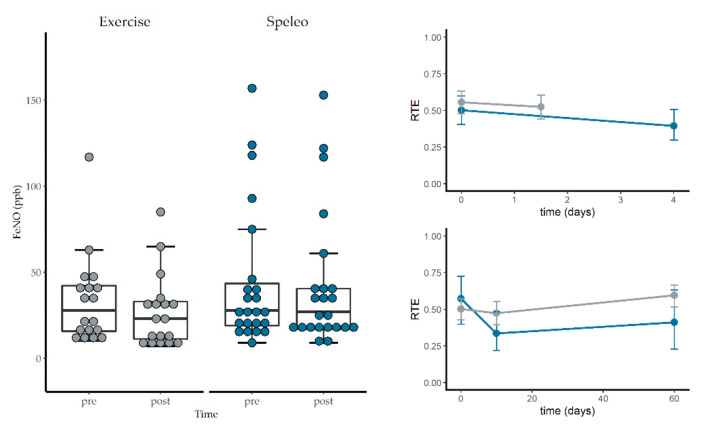
Short term effects of winter exercise on fractional exhaled nitric oxide. Exercise group is colored grey and speleotherapy group is colored blue. Upper right plot shows the relative treatment effects of the short-term measurements of FeNO. The lower right plot shows the relative treatment effects of FeNO over all time points (day 0–day 60). Error bars represent upper and lower confidence interval of the relative treatment effects (RTEs).

The analysis of variance type test for FeNO and nasal NO revealed no significant main effects for treatment, time, or the interaction. However, the RTEs indicate a decrease of FeNO in the exercise group (see Figure 4). No significant effects were found for any spirometry parameter, except for a time effect for FEV_1_/FVC (%) but post hoc test did not reveal any interaction effects at the single time points (see Table 3).

### 3.4. Short-Term Effects of Winter Hiking and Speleotherapy

The F1-LD-F1-model for the short-term effects on FeNO revealed a significant time (F_(1.00, ∞)_ = 27.77, *p* < 0.001) and interaction effect (F_(1.00, ∞)_ = 7.58, *p* = 0.01; RTE speleotherapy group: 0.56, 0.52 pre and post speleotherapy, RTE exercise group: 0.50, 0.39 pre and post winter hike). No significant treatment effect was found (F_(1.00, ∞)_ = 1.03, *p* = 0.31). Relative treatment effects indicate a stronger decrease of FeNO in the exercise group (see Figure 4).

### 3.5. RhinAsthma Quality of Life Questionnaire (German Adapted Version)

The analysis of variance type test for the total score of the German version of the RhinAsthma quality of Life questionnaire revealed a significant main effect for time, but post-hoc tests did not show any interaction effects at the single time points. RTEs indicate an improvement of allergic symptoms in both groups. No significant treatment or interaction effects were found for the subscales. Significant time effects were found for all subscales, except for “impairment in sensory perception” (see Table 4).

### 3.6. Nasal Eosinophilic Count and Muco-ciliary Clearance Time

**Figure 5:**
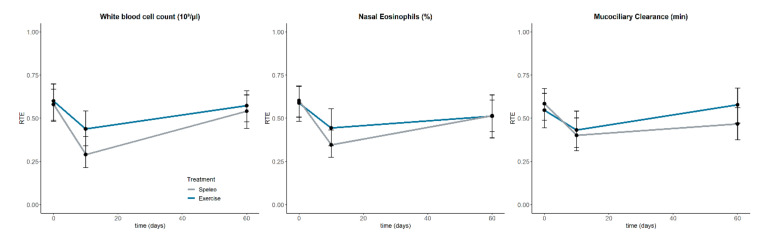
Relative treatment effects (RTE) of the white blood cell count (**Left**), the nasal eosinophilic cell count (middle) and the muco-ciliary clearance time (**Right**). Error bars represent upper and lower confidence interval of the RTEs.

No significant treatment or interaction effects were found for the nasal eosinophilic cell count and the muco-ciliary clearance time. Although, significant time effects were found, post hoc tests did not reveal any interaction effects at the single time points. The RTEs indicate a short-term improvement of nasal eosinophilic cell count and the muco-ciliary clearance time in both groups (see Table 3, Figure 5).

### 3.7. Differential Blood Count

No significant treatment or interaction effects were found for the differential blood count. A significant time effect was found for the white blood cell count (WBC) but post hoc test did not reveal any interaction effects at the single time points. However, the RTEs indicate a decrease of the WBC in both groups, with the speleotherapy group showing a stronger decrease (see Figure 5). The eosinophilic blood count presents with a trend for the main interaction effect. Although, RTEs indicate a baseline difference, no significant difference was found (t_(26.4)_ = 1.59, *p* = 0.12). Furthermore, RTEs indicate an increase in eosinophilic blood count in the speleotherapy group (see Table 3).

### 3.8. Six-Minute Walk Test

The speleotherapy group is characterized by a better baseline performance in the predicted 6MWT-distance in comparison to the exercise group (t_(37.1)_ = −2.26, *p* = 0.03; see Table 2). The F1-LD-F1-model for the predicted 6MWT-distance revealed a significant main effect for treatment and time. Post hoc tests yielded significant treatment and time effects at day 10 (F_(1.00, ∞)_ = 10.06, *p* < 0.009) and day 60 (F_(1.00, ∞)_ = 6.05, *p* < 0.056). The peak minute ventilation (PVE) also indicates a baseline difference (t_(37.5)_ = −2.01, *p* = 0.05). Again, the speleotherapy group is presenting with higher levels (61.89 ± 15.02 l/min) in comparison to the exercise group (52.67 ± 14.27 l/min). Significant main effects for treatment and time were found and post hoc test revealed a significant treatment effect at day 10 (F_(1.00, ∞)_ = 9.50, *p* < 0.012). RTEs indicate an increase in the PVE in the speleotherapy group. Significant time effects were found for the BORG-fatigue scale but post hoc test did not reveal any interaction effects at the single time points. No significant effects were found for the peak respiratory frequency and the BORG-dyspnea scale (see Table 5).

### 3.9. Visual Analogue Scale

For the inverse visual analogue scales for allergic symptoms and general health status, F1-LD-F1-model revealed significant time effects, indicating an improvement in both groups. Post hoc test shows a trend in the interaction effect at day 60, indicating a faster return to baseline within the speleotherapy group (see Table 4).

### 3.10. Sample Size Simulation

**Figure 6:**
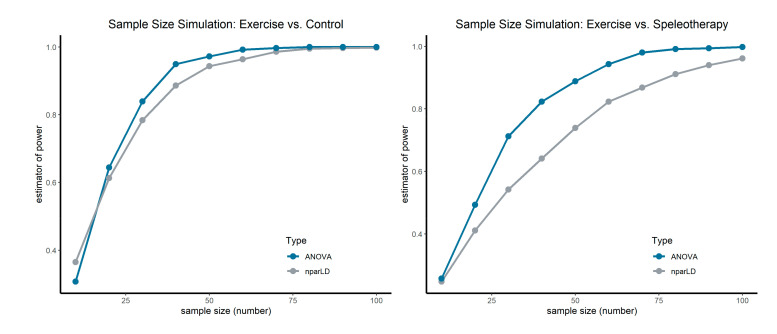
Sample size simulation for the primary outcome FeNO. Left graph shows the comparison of the exercise group vs. the control group. Right graph shows the comparison of the exercise and speleotherapy group. Sample size simulation was performed for nparLD and ANOVA.

According to the bootstrap sample size simulation a sample size of at least n = 40 per group is needed to reach a power of 1 − β = 0.89 in the case of exercise vs. control (see Figure 6). The comparison of the exercise and speleotherapy group requires larger sample sizes—a sample size of at least n = 70 per group is needed to reach a power of 1 − β = 0.87. Detailed results from the sample size simulation can be found in Table S4 (Exercise vs. Control) and Table S5 (Exercise vs. Speleotherapy).

## 4. Discussion

Although speleotherapy is a widely accepted additional treatment option for chronic respiratory diseases, the existing evidence is rather low [21]. In this randomized controlled clinical trial, we investigated the effects of speleotherapy in combination with recreational winter exercise on patients with allergic rhinitis and/or asthma.

Fractional exhaled NO is a reliable surrogate parameter to measure allergic airway inflammation [55]. In sedentary adults with asthma, moderate-intensity exercise is associated with a decrease in FeNO [56]. Although both study groups participated in recreational winter exercise activities, no significant treatment, time or interaction effects were observed for FeNO and nasal NO. However, when focusing on the relative treatment effects, a decrease of FeNO can be observed in the exercise group (Ex. × T0 = 0.57, Ex. × T1 = 0.34) in comparison to the speleotherapy group (Sp. × T0 = 0.50, Sp. × T1 = 0.47). A similar picture emerges for the short-term effects of recreational winter exercise and speleotherapy: a significant interaction effect in combination with the decreasing RTEs in the exercise group indicate a greater impact of recreational winter exercise on FeNO in comparison to a single speleotherapy session. However, one must keep in mind, that the time factor between the exercise and speleotherapy group was quite different (4 h winter hiking vs. 1.5 h speleotherapy). Full day winter exercise seems to have a stronger impact on allergic airway inflammation than winter exercise in combination with speleotherapy. When looking on the sample size simulation, a sample size of n = 70 per group would have been needed to reveal a statistically significant difference of the FeNO levels between winter exercise and winter exercise in combination with speleotherapy.

As a patient-centered outcome, allergy and asthma specific quality of life was assessed. The results from the RhinAsthma quality of life questionnaire show significant improvements in quality of life in both groups. The inverse visual analogue scales for allergic symptoms and general health status also indicate improvements in both groups. However, post hoc test shows a trend in the interaction effect at day 60 for allergic symptoms, revealing a faster return to baseline within the speleotherapy group. Significant time effects were also found for the nasal eosinophilic cell count and the muco-ciliary clearance time, thus indicating comparable improvements in both groups. Although, the muco-ciliary clearance time improves in both groups, the RTEs only suggest a sustainable effect in the speleotherapy group. Furthermore, the RTEs indicate a stronger improvement of the nasal eosinophilic cell count in the speleotherapy group, but statistical significance is missing. Changes in the differential blood count are more evident in the speleotherapy group than in the exercise group. In both groups the white blood cell count is decreasing, but this effect is more evident in the speleotherapy group. Furthermore, a trend for the interaction effect can be observed for the eosinophilic blood cell count, which seems to increase in the speleotherapy group. No relevant changes were found for spirometry parameters, but most participants started with a relatively good spirometry performance, as was already the case in the first part of the WESPAA study [9]. However, an improvement of normative values through a ten-day intervention can be hardly expected. In a study population with more severe limitations in lung function, an improvement in spirometry parameters could have been possible. For the six-minute walk test, no significant differences between the speleotherapy and exercise group were found. The observed treatment effects for the predicted six-minute walk distance and the peak minute ventilation originate from a significant baseline difference. Most participants present with a relatively good performance for the six-minute walk test at baseline. The short intervention period of ten days may be too short to induce sustainable improvements in the cardio-respiratory fitness level.

The specific microclimate of caves and mines is complex and offers many variations. This may be one reason, why the specific curative factors of speleotherapy are not yet fully understood. Due to the complexity of speleotherapy, it is challenging to separate specific from non-specific effects. The speleotherapy location in the WESPAA-Study is characterized by constant moderate low temperature (7.45–8.02 °C), high relative humidity, low fine dust concentrations and slightly higher natural radiation. Existing speleotherapy proposes that the combination of high relative humidity and low temperature improve muco-ciliary clearance [23]. However, the muco-ciliary clearance rate also improves in the winter exercise group. During the evaluation of short-term effects of winter exercise and speleotherapy, muco-ciliary clearance rates should have been measured, to get more insight of the specific effects of winter exercise and speleotherapy. The temperature range of the used speleotherapy location does not represent any acute trigger for bronchoconstriction [57]. Furthermore, the low fine dust concentration and the absence of allergy triggers could be a possible curative factor [58]. Mines and caves are often characterized by higher natural radiation levels. Indoor radon gas concentration limits in Austria are set at 400 Bq/m^3^ [59]. At the speleotherapy location of the WESPAA-Study more than twice as much radon concentration levels are present. The measured radon gas concentration of 954.32 Bq/m^3^ corresponds to a dose of 4.77 μSv per hour exposure (equilibrium ratio between progeny and parental radon was assumed to be 0.5). During the intervention, the participants were exposed to a total dose of 42.95 μSv, which is a negligibly small dose and is described to have no biological effects. Therefore, low dose radiation effects from radon can be excluded as a possible influencing factor in the “Schaubergwerk Hochfeld”. However, no other sources of natural radiation such as ^40^K (potassium radioisotope) were measured, so effects of higher natural radiation levels in general cannot be fully excluded.

Regular physical activity is not only associated with positive health benefits in the general population, but also people with respiratory allergies benefit from physical activity [60,61,62]. Although asthma is more common among athletes [63], evidence based concepts are needed for recreational winter exercise, as adequate self-management of allergic respiratory diseases should include physical activity. Therefore, no speleotherapy group without winter exercise was included in the WESPAA-Study. All observed effects in the speleotherapy group are therefore combined winter exercise effects. The results from the first part of the WESPAA-Study clearly support the relevance of exercise for allergic respiratory diseases. Recreational outdoor winter exercise reduces allergic airway inflammation and improves allergic symptoms [9]. No undesirable effects like bronchoconstriction or respiratory distress were observed in any group, although exercising in cold air may cause bronchoconstriction [64]. However, the Holiday Region Hohe Tauern National Park offers perfect conditions with good environmental air quality [65,66]. Both, recreational winter exercise and speleotherapy may be recommended to patients with good disease control and adequate self-management.

### Limitations

Due to limited resources and a high supervisor-participant ratio during the exercise program, it was not possible to conduct both intervention groups simultaneously. Although environmental parameters are quite comparable in March and December, this season gap should be kept in mind, when interpreting the results, as the indoor allergen load may vary. No speleotherapy group without winter exercise was included in the WESPAA-Study. All observed effects in the speleotherapy group are therefore combined winter exercise effects. A parallel control group without any intervention but with accommodation at the same site, would have been a valuable extension of the study design. Because of limited resources it was not possible to include an onsite control group. The intensity and duration of the exercise program was significantly different between the two intervention groups. To compensate for the small sample size, all statistical analyses were performed using rank-based nonparametric methods with ANOVA-type statistics to accurately control the type I error rate. Within this small study population, it is not possible to represent the full disease severity spectrum of allergic rhinitis and/or asthma. The study population presented with good baseline spirometry and six-minute walk test performance. The results are therefore limited for highly functioning patients with good disease control. However, further studies are needed for detailed recommendations on speleotherapy and outdoor winter exercise for people with allergic rhinitis and/or asthma. The sample size simulation revealed that a sample size of n = 70 per group is needed for the comparison of the speleotherapy and exercise group.

## 5. Conclusions

A ten-day intervention with speleotherapy and/or winter exercise improves allergic symptoms and health related quality of life in adults with allergic rhinitis and/or asthma. No participant experienced any unexpected or adverse effects during the winter sports and speleotherapy activities. Recreational winter exercise and/or speleotherapy may be therefore recommended for highly functioning patients with good diseases control. In the future, management of chronic disease, winter exercise and/or speleotherapy could add a new chapter of patient-centered disease management. However, further studies with larger sample sizes are required to investigate the specific effects of speleotherapy and winter exercise.

## Figures and Tables

**Table 1:** Baseline characteristics of the study population.

Baseline Variables	Exercise Group (n = 18)		Speleotherapy Group (n = 23)		Baseline Tests	
	Mean ± SD	Median ± IQR	Mean ± SD	Median ± IQR		
Gender	male n = 5	female n = 13	male n = 12	female n = 11	0.21	χ^2^ Test
Residence+	rural n = 5	urban n = 13	rural n = 6	urban n = 17	0.67	χ^2^ Test
RAST/PRICK	2 or + n = 8	>3 or > ++ n = 10	2 or + n = 7	>3 or > ++ n = 16	0.52	χ^2^ Test
Age (years)	40.61 ± 12.12	45.0 ± 22.25	32.78 ± 9.90	29.00 ± 14.00	0.03 *	T-Test
Height (m)	172.61 ± 6.66	172.0 ± 7.50	174.35 ± 9.10	175.00 ± 12.50	0.48	T-Test
Weight (kg)	69.83 ± 9.83	69.0 ± 10.00	68.39 ± 16.24	70.00 ± 15.00	0.73	T-Test
Pulse (bpm)	70.99 ± 12.57	70.38 ± 23.00	72.91 ± 13.30	70.00 ± 19.50	0.64	T-Test
BP-Systole (mmHg)	125.28 ± 14.46	123.5 ± 14.50	121.48 ± 11.29	124.00 ± 9.50	0.48	U-Test
BP-Diastole (mmHg)	76.44 ± 9.97	78.0 ± 15.00	74.74 ± 10.46	75.00 ± 15.50	0.60	T-Test
ACT (score)	21.39 ± 3.57	22.5 ± 4.50	22.17 ± 3.04	23.00 ± 3.50	0.54	U-Test
FeNO (ppb)	40.78 ± 23.03	38.0 ± 39.00	36.91 ± 28.26	28.00 ± 23.00	0.69	T-Test
FEV_1_ (%)	96.28 ± 20.98	98.5 ± 21.25	101.39 ± 17.78	104.00 ± 14.00	0.38	U-Test
FVC (%)	108.17 ± 17.66	108.0 ± 12.75	107.22 ± 14.48	107.00 ± 12.50	0.78	U-Test
FEV_1_/FVC (%)	93.39 ± 11.81	95.0 ± 17.00	97.43 ± 9.26	99.00 ± 9.50	0.20	U-Test
6MWT-Distance (%)	104.35 ± 8.17	105.99 ± 10.97	112.01 ± 13.36	111.74 ± 21.38	0.03 *	T-Test

+ number of participants living in an urban/rural environment; urban >10,000 citizens. ++ is threating system of the PRICK test; IQR, interquartile range; RAST, radio-allergo-sorbent test; PRICK, allergy-prick test; BP, blood pressure; ACT, asthma control test; FEV_1_, forced expiratory volume in 1 s; FVC, forced expiratory volume; FEV_1_/FVC, forced expiratory volume in 1 s of forced vital capacity; 6MWT, six-minute walk test. *< 0.05.

**Table 2:** Characteristics of the exercise program.

Timepoint	Exercise Group (n = 18)			Speleotherapy Group (n = 23)		
Day	Type	Distance (km)	Altitude (m)	Type	Distance (km)	Altitude (m)
day 1	winter hiking	9.9 km	557 m	winter hiking	10.0 km	400 m
day 2	alpine skiing	42.8 km	-	alpine skiing	23.2 km	-
day 3	winter hiking	15.3 km	448 m	cross-country skiing	5.4 km	60 m
day 5	alpine skiing	38.7 km	-	winter hiking	8.1 km	335 m
day 6	winter hiking	5.9 km	258 m	winter hiking	2.5 km	100 m
day 7	alpine skiing	55.8 km	-	winter hiking	8.2 km	217 m
day 8	alpine skiing	31.0 km	-	alpine skiing	34 km	-
day 9	winter hiking	13.4 km	381 m	winter hiking	7.0 km	248 m
day 1–10	average hiking	11.1 km	411 m	average hiking	6.9 km	226.7 m
day 1–10	average skiing	42.1 km	-	average skiing	28.6 km	-

**Table 3:** Results from the F1-LD-F1 model reporting time, treatment, and interaction effects. Abbreviations: Sp = speleotherapy, Ex = winter exercise, T0 = day 0, T1 = day 10, T2 = day 60, FeNO = fractional exhaled nitric oxide, FEV_1_/FVC = forced expiratory volume in 1 s of forced vital capacity, MEF_25–75%_ = mid-expiratory flow at 25–75% of the FVC, PEF = peak expiratory flow, RBC = red blood cell count, WBC = white blood cell count.

Parameter	F1-LD-F1 Model				Relative Treatment Effects (RTE)					
		F	*p*-Value		Time		Speleotheapy		Winter Exercise	
FeNO							Speleo	0.52	Exercise	0.44
	Treat	0.65 (1.00, ∞)	1.000	n.s	T0	0.54	Sp. × T0	0.50	Ex. × T0	0.57
	Time	4.06 (1.37, ∞)	0.093	n.s	T1	0.40	Sp. × T1	0.47	Ex. × T1	0.34
	Treat × Time	3.93 (1.37, ∞)	0.103	n.s	T2	0.50	Sp. × T2	0.60	Ex. × T2	0.41
Nasal NO							Speleo	0.51	Exercise	0.47
	Treat	0.21 (1.00, ∞)	1.000	n.s	T0	0.52	Sp. × T0	0.49	Ex. × T0	0.55
	Time	0.33 (1.83, ∞)	1.000	n.s	T1	0.46	Sp. × T1	0.50	Ex. × T1	0.42
	Treat × Time	0.76 (1.83, ∞)	0.733	n.s	T2	0.49	Sp. × T2	0.54	Ex. × T2	0.44
Nasal eosinophils (%)							Speleo	0.49	Exercise	0.52
	Treat	0.13 (1.00, ∞)	1.000	n.s	T0	0.60	Sp. × T0	0.60	Ex. × T0	0.59
	Time	11.76 (1.73, ∞)	<0.001	***	T1	0.40	Sp. × T1	0.35	Ex. × T1	0.44
	Treat × Time	1.10 (1.73, ∞)	0.654	n.s	T2	0.51	Sp. × T2	0.52	Ex. × T2	0.51
Mucociliary clearance (min)							Speleo	0.48	Exercise	0.52
	Treat	0.23 (1.00, ∞)	1.000	n.s	T0	0.57	Sp. × T0	0.59	Ex. × T0	0.55
	Time	6.78 (1.84, ∞)	0.006	**	T1	0.42	Sp. × T1	0.40	Ex. × T1	0.43
	Treat × Time	1.58 (1.84, ∞)	0.470	n.s	T2	0.52	Sp. × T2	0.47	Ex. × T2	0.58
FVC (%)							Speleo	0.48	Exercise	0.53
	Treat	0.28 (1.00, ∞)	1.000	n.s	T0	0.48	Sp. × T0	0.47	Ex. × T0	0.50
	Time	0.23 (1.90, ∞)	0.220	n.s	T1	0.50	Sp. × T1	0.48	Ex. × T1	0.52
	Treat × Time	0.33 (1.90, ∞)	0.705	n.s	T2	0.52	Sp. × T2	0.49	Ex. × T2	0.56
FEV_1_ (%)							Speleo	0.53	Exercise	0.46
	Treat	0.60 (1.00, ∞)	0.689	n.s	T0	0.49	Sp. × T0	0.53	Ex. × T0	0.45
	Time	1.04 (1.91, ∞)	0.416	n.s	T1	0.51	Sp. × T1	0.54	Ex. × T1	0.48
	Treat × Time	0.51 (1.91, ∞)	1.000	n.s	T2	0.49	Sp. × T2	0.52	Ex. × T2	0.46
FEV_1_/FVC (%)							Speleo	0.54	Exercise	0.44
	Treat	1.24 (1.00, ∞)	0.585	n.s	T0	0.50	Sp. × T0	0.56	Ex. × T0	0.44
	Time	6.72 (1.76, ∞)	0.008	**	T1	0.52	Sp. × T1	0.55	Ex. × T1	0.48
	Treat × Time	1.81 (1.76, ∞)	0.504	n.s	T2	0.46	Sp. × T2	0.52	Ex. × T2	0.41
PEF (%)							Speleo	0.53	Exercise	0.46
	Treat	0.58 (1.00, ∞)	1.000	n.s	T0	0.47	Sp. × T0	0.47	Ex. × T0	0.46
	Time	2.08 (1.95, ∞)	0.253	n.s	T1	0.50	Sp. × T1	0.55	Ex. × T1	0.44
	Treat × Time	1.77 (1.95, ∞)	0.326	n.s	T2	0.53	Sp. × T2	0.57	Ex. × T2	0.48
MEF_25–75%_ (%)							Speleo	0.55	Exercise	0.43
	Treat	1.93 (1.00, ∞)	0.501	n.s	T0	0.49	Sp. × T0	0.56	Ex. × T0	0.42
	Time	3.64 (1.92, ∞)	0.112	n.s	T1	0.51	Sp. × T1	0.56	Ex. × T1	0.47
	Treat × Time	2.28 (1.92, ∞)	0.314	n.s	T2	0.48	Sp. × T2	0.55	Ex. × T2	0.41
WBC (10^3^/µL)							Speleo	0.47	Exercise	0.54
	Treat	0.94 (1.00, ∞)	0.791	n.s	T0	0.59	Sp. × T0	0.58	Ex. × T0	0.60
	Time	16.01 (1.89, ∞)	<0.001	***	T1	0.36	Sp. × T1	0.29	Ex. × T1	0.44
	Treat × Time	1.35 (1.89, ∞)	0.518	n.s	T2	0.56	Sp. × T2	0.54	Ex. × T2	0.57
RBC (10^6^/µL)							Speleo	0.54	Exercise	0.45
	Treat	0.99 (1.00, ∞)	0.642	n.s	T0	0.51	Sp. × T0	0.54	Ex. × T0	0.47
	Time	0.22 (1.86, ∞)	0.783	n.s	T1	0.49	Sp. × T1	0.54	Ex. × T1	0.45
	Treat × Time	0.06 (1.86, ∞)	1.000	n.s	T2	0.49	Sp. × T2	0.53	Ex. × T2	0.44
Neutrophil granulocytes (%)							Speleo	0.51	Exercise	0.49
	Treat	0.08 (1.00, ∞)	0.837	n.s	T0	0.54	Sp. × T0	0.55	Ex. × T0	0.54
	Time	2.47 (1.77, ∞)	0.184	n.s	T1	0.45	Sp. × T1	0.43	Ex. × T1	0.47
	Treat × Time	1.12 (1.77, ∞)	0.321	n.s	T2	0.50	Sp. × T2	0.54	Ex. × T2	0.46
Eosinophil granulocytes (%)							Speleo	0.47	Exercise	0.54
	Treat	0.61 (1.00, ∞)	0.872	n.s	T0	0.48	Sp. × T0	0.40	Ex. × T0	0.56
	Time	2.25 (2.00, ∞)	0.214	n.s	T1	0.55	Sp. × T1	0.56	Ex. × T1	0.54
	Treat × Time	3.38 (2.00, ∞)	0.070		T2	0.49	Sp. × T2	0.46	Ex. × T2	0.51

*** < 0.001; ** < 0.01; <0.1; n.s. not significant.

**Table 4:** Results from the F1-LD-F1 model reporting time, treatment, and interaction effects. Abbreviations: Sp = speleotherapy, Ex = winter exercise, T0 = day 0, T1 = day 10, T2 = day 60, 6MWT = six-minute walk test.

Parameter	F1-LD-F1 Model				Relative Treatment Effects (RTE)					
		F	*p*-Value		Time		Speleotheapy		Winter Exercise	
6MWT Distance (%)							Speleo	0.60	Exercise	0.37
	Treat	9.59 (1.00, ∞)	0.010	*	T0	0.43	Sp. × T0	0.53	Ex. × T0	0.34
	Time	5.04 (1.95, ∞)	0.019	*	T1	0.53	Sp. × T1	0.68	Ex. × T1	0.39
	Treat × Time	1.50 (1.95, ∞)	1.000	n.s	T2	0.49	Sp. × T2	0.59	Ex. × T2	0.39
Peak respiratory							Speleo	0.50	Exercise	0.50
frequency (L/min)	Treat	0.00 (1.00, ∞)	1.000	n.s	T0	0.50	Sp. × T0	0.46	Ex. × T0	0.54
	Time	0.06 (1.83, ∞)	1.000	n.s	T1	0.50	Sp. × T1	0.52	Ex. × T1	0.49
	Treat × Time	1.42 (1.83, ∞)	0.449	n.s	T2	0.49	Sp. × T2	0.51	Ex. × T2	0.47
Peak minute							Speleo	0.60	Exercise	0.38
ventilation (L/min)	Treat	8.51 (1.00, ∞)	0.018	*	T0	0.44	Sp. × T0	0.53	Ex. × T0	0.35
	Time	5.81 (1.80, ∞)	0.018	*	T1	0.55	Sp. × T1	0.70	Ex. × T1	0.40
	Treat × Time	2.21 (1.80, ∞)	0.579	n.s	T2	0.47	Sp. × T2	0.56	Ex. × T2	0.38
BORG Dyspnea							Speleo	0.48	Exercise	0.52
pre (score)	Treat	0.35 (1.00, ∞)	0.725	n.s	T0	0.54	Sp. × T0	0.51	Ex. × T0	0.57
	Time	2.42 (1.66, ∞)	0.198	n.s	T1	0.45	Sp. × T1	0.42	Ex. × T1	0.48
	Treat × Time	0.28 (1.66, ∞)	0.841	n.s	T2	0.52	Sp. × T2	0.51	Ex. × T2	0.52
BORG Dyspnea post							Speleo	0.55	Exercise	0.44
(score)	Treat	1.84 (1.00, ∞)	0.876	n.s	T0	0.54	Sp. × T0	0.56	Ex. × T0	0.52
	Time	2.66 (1.80, ∞)	0.229	n.s	T1	0.46	Sp. × T1	0.50	Ex. × T1	0.43
	Treat × Time	2.70 (1.80, ∞)	0.376	n.s	T2	0.48	Sp. × T2	0.58	Ex. × T2	0.38
BORG Fatigue pre							Speleo	0.52	Exercise	0.48
(score)	Treat	0.31 (1.00, ∞)	1.000	n.s	T0	0.60	Sp. × T0	0.62	Ex. × T0	0.57
	Time	10.60 (1.89, ∞)	<0.001	***	T1	0.41	Sp. × T1	0.40	Ex. × T1	0.43
	Treat × Time	1.16 (1.89, ∞)	1.000	n.s	T2	0.49	Sp. × T2	0.53	Ex. × T2	0.44
BORG Fatigue post							Speleo	0.54	Exercise	0.45
(score)	Treat	1.46 (1.00, ∞)	1.000	n.s	T0	0.56	Sp. × T0	0.61	Ex. × T0	0.51
	Time	6.81 (1.94, ∞)	0.006	**	T1	0.49	Sp. × T1	0.53	Ex. × T1	0.45
	Treat × Time	0.08 (1.94, ∞)	1.000	n.s	T2	0.44	Sp. x T2	0.49	Ex. × T2	0.39

*** < 0.001; ** < 0.01; * < 0.05; < 0.1; n.s. not significant.

**Table 5:** Results from the F1-LD-F1 model reporting time, treatment, and interaction effects. Abbreviations: Sp = speleotherapy, Ex = winter exercise, T0 = day 0, T1 = day 10, T2 = day 60, VAS = visual analogue scale.

Parameter	F1-LD-F1 Model				Relative Treatment Effects (RTE)					
		F	*p* Value		Time		Speleotheapy		Winter Exercise	
RhinAsthma Total score (score)							Speleo	0.53	Exercise	0.46
	Treat	0.64 (1.00, ∞)	1.000	n.s	T0	0.59	Sp. × T0	0.64	Ex. × T0	0.55
	Time	12.52 (1.86, ∞)	<0.001	***	T1	0.45	Sp. × T1	0.44	Ex. × T1	0.45
	Treat × Time	1.68 (1.86, ∞)	0.566	n.s	T2	0.45	Sp. × T2	0.50	Ex. × T2	0.40
RhinAshtma Limitation in daily life (score)							Speleo	0.54	Exercise	0.45
	Treat	1.25 (1.00, ∞)	1.000	n.s	T0	0.58	Sp. × T0	0.66	Ex. × T0	0.51
	Time	12.07 (1.90 ∞)	<0.001	***	T1	0.47	Sp. × T1	0.50	Ex. × T1	0.43
	Treat × Time	1.08 (1.90, ∞)	1.000	n.s	T2	0.43	Sp. × T2	0.46	Ex. × T2	0.40
RhinAsthma Respiratory problems (score)							Speleo	0.50	Exercise	0.50
	Treat	0.00 (1.00, ∞)	1.000	n.s	T0	0.61	Sp. × T0	0.63	Ex. × T0	0.58
	Time	10.99 (1.94, ∞)	<0.001	***	T1	0.44	Sp. × T1	0.41	Ex. × T1	0.46
	Treat × Time	0.96 (1.94, ∞)	1.000	n.s	T2	0.46	Sp. × T2	0.45	Ex. × T2	0.47
RhinAsthma Rhino-conjunctivitis score (score)							Speleo	0.52	Exercise	0.48
	Treat	0.32 (1.00, ∞)	1.000	n.s	T0	0.58	Sp. × T0	0.58	Ex. × T0	0.57
	Time	9.36 (1.94, ∞)	<0.001	***	T1	0.39	Sp. × T1	0.37	Ex. × T1	0.41
	Treat × Time	2.23 (1.94, ∞)	0.328	n.s	T2	0.53	Sp. × T2	0.60	Ex. × T2	0.45
RhinAsthma Treatment and med. problems (score)							Speleo	0.51	Exercise	0.48
	Treat	0.18 (1.00, ∞)	1.000	n.s	T0	0.59	Sp. × T0	0.61	Ex. × T0	0.56
	Time	11.57 (1.88, ∞)	<0.001	***	T1	0.49	Sp. × T1	0.51	Ex. × T1	0.47
	Treat × Time	0.21 (1.88, ∞)	1.000	n.s	T2	0.42	Sp. × T2	0.42	Ex. × T2	0.41
RhinAsthma Impairment in sensory percept. (score)							Speleo	0.51	Exercise	0.48
	Treat	0.12 (1.00, ∞)	1.000	n.s.	T0	0.55	Sp. × T0	0.58	Ex. × T0	0.51
	Time	3.30 (1.88, ∞)	0.199	n.s.	T1	0.49	Sp. × T1	0.47	Ex. × T1	0.50
	Treat × Time	1.12 (1.88, ∞)	0.648	n.s.	T2	0.46	Sp. × T2	0.49	Ex. × T2	0.43
							Speleo	0.50	Exercise	0.50
VAS Allergy (score)	Treat	0.01 (1.00, ∞)	1.000	n.s	T0	0.40	Sp. × T0	0.45	Ex. × T0	0.36
	Time	13.41 (1.98, ∞)	<0.001	***	T1	0.62	Sp. × T1	0.61	Ex. × T1	0.62
	Treat × Time	2.68 (1.98, ∞)	0.138	n.s	T2	0.48	Sp. × T2	0.43	Ex. × T2	0.53
	Treat × T2	5.17 (1.00, ∞)	0.069							
VAS Health Status (score)							Speleo	0.52	Exercise	0.48
	Treat	0.33 (1.00, ∞)	1.000	n.s	T0	0.37	Sp. × T0	0.40	Ex. × T0	0.33
	Time	16.01 (1.56, ∞)	<0.001	***	T1	0.60	Sp. × T1	0.62	Ex. × T1	0.57
	Treat × Time	0.30 (1.56, ∞)	1.000	n.s	T2	0.53	Sp. × T2	0.53	Ex. × T2	0.53

*** < 0.001; n.s. not significant.

## Supplementary Materials

The following are available online at https://www.mdpi.com/2077-0383/9/10/3311/s1, Table S1: Descriptive statistics of the speleotherapy group. Table S2: Descriptive statistics of the exercise group. Table S3: Descriptive statistics of the control group. Table S4: Sample Size Simulation Exercise vs. Control group. Table S5: Sample Size simulation Exercise vs. Speleotherapy group.

## Abbreviations

ATS/ETS	American thoracic society/European respiratory society
FeNO	Fractional exhaled nitric oxide
FEV_1_	Forced expiratory volume in 1 s
FEV_1_/FVC	Forced expiratory volume in 1 s of forced vital capacity
FVC	Forced expiratory volume
IgE	Immunoglobulin E
MEF_25–75%_	Mid-expiratory flow at 25–75% of the FVC
nparLD	Nonparametric longitudinal data analysis
PEF	Peak expiratory flow
PVE	Peak minute ventilation
RAST	Radio-allergo-sorbent-test
RBC	Red blood cell count
RTE	Relative treatment effect
VAS	Visual analogue scale
WBC	White blood cell count
^222^Rn	Radon radioisotope
^40^K	Potassium radioisotope
6MWT	Six-minute walk test
