# Supplementary material for: Winter Exercise and Speleotherapy for Allergy and Asthma: A Randomized Controlled Clinical Trial
Source: J Clin Med. 2020 Oct 15;9(10):3311. doi: 10.3390/jcm9103311 (PMC7602599; doi:10.3390/jcm9103311)
Supplement: Supplementary file 1 [file jcm-09-03311-s001.zip › Supplemental Descriptive Statistics.docx]

Table 1: Speleo-Group

day 0 day 10 day 60

Speleo-Group Mean SD Median IQR Mean SD Median IQR Mean SD Median IQR

**Physiology**

| Pulse (bpm) | 72.91 | 13.30 | 70.00 | 19.50 | 73.17 | 5.12 | 74.00 | 5.50 | 69.96 | 11.49 | 68.00 | 9.00 |
| --- | --- | --- | --- | --- | --- | --- | --- | --- | --- | --- | --- | --- |
| BP-Systole (mmHg) | 121.48 | 11.29 | 124.00 | 9.50 | 120.43 | 11.87 | 119.00 | 16.00 | 117.61 | 12.93 | 117.00 | 11.00 |
| BP-Diastole (mmHg) | 74.74 | 10.46 | 75.00 | 15.50 | 71.52 | 9.69 | 73.00 | 12.00 | 70.13 | 6.64 | 68.00 | 7.00 |
| FeNO oral (ppb) | 36.91 | 28.26 | 28.00 | 23.00 | 39.96 | 37.84 | 25.00 | 20.50 | 47.52 | 36.09 | 38.00 | 32.50 |
| FeNO nasal (ppb) | 427.57 | 259.62 | 374.00 | 317.00 | 418.61 | 188.10 | 401.00 | 265.50 | 462.91 | 235.88 | 470.00 | 248.50 |
| Nasal eosinophilic count (%) | 4.36 | 4.47 | 2.69 | 3.49 | 1.51 | 1.62 | 0.92 | 1.70 | 2.83 | 2.74 | 2.60 | 2.75 |
| Mucociliary Clearance (min) | 12.25 | 6.00 | 15.00 | 7.43 | 8.20 | 4.97 | 7.56 | 8.75 | 9.41 | 5.24 | 9.00 | 10.80 |

**Spirometry (%)**

| FVC | 107.22 | 14.48 | 107.00 | 12.50 | 108.70 | 13.38 | 109.00 | 18.50 | 108.39 | 14.85 | 108.00 | 15.50 |
| --- | --- | --- | --- | --- | --- | --- | --- | --- | --- | --- | --- | --- |
| FEV1 | 101.39 | 17.78 | 104.00 | 14.00 | 102.61 | 15.83 | 102.00 | 13.50 | 101.43 | 19.23 | 103.00 | 18.50 |
| FEV1/FVC | 97.43 | 9.26 | 99.00 | 9.50 | 97.48 | 9.02 | 98.00 | 12.00 | 96.13 | 11.12 | 98.00 | 11.50 |
| PEF | 101.43 | 13.78 | 103.00 | 13.50 | 106.57 | 15.26 | 104.00 | 25.00 | 107.52 | 16.65 | 108.00 | 20.00 |
| MEF25-75% | 86.70 | 30.72 | 87.00 | 29.00 | 87.22 | 30.10 | 90.00 | 32.50 | 85.30 | 33.12 | 86.00 | 31.50 |

**Differential Blood Count**

| WBC (103µl) | 7.39 | 1.88 | 6.38 | 2.76 | 5.53 | 1.55 | 5.03 | 1.99 | 6.92 | 1.44 | 7.05 | 1.95 |
| --- | --- | --- | --- | --- | --- | --- | --- | --- | --- | --- | --- | --- |
| RBC (106µl) | 4.89 | 0.42 | 5.01 | 0.74 | 4.90 | 0.41 | 4.94 | 0.67 | 4.91 | 0.49 | 4.91 | 0.86 |
| Blood neutrophil count (%) | 60.48 | 8.07 | 59.60 | 13.00 | 57.17 | 6.38 | 56.00 | 5.35 | 59.70 | 6.50 | 60.90 | 9.20 |
| Blood eosinophil count (%) | 2.59 | 1.78 | 2.20 | 1.35 | 3.89 | 3.11 | 3.10 | 2.50 | 3.18 | 2.64 | 2.40 | 1.70 |
| **Minute Walk Test** |  |  |  |  |  |  |  |  |  |  |  |  |
| Distance (%) | 112.01 | 13.36 | 111.74 | 21.38 | 117.98 | 11.88 | 117.41 | 18.30 | 113.82 | 10.98 | 113.36 | 15.79 |
| Peak respiratory frequency | 34.27 | 10.95 | 32.90 | 12.15 | 34.92 | 8.09 | 32.90 | 11.60 | 34.46 | 8.46 | 34.50 | 8.45 |
| Peak minute ventilation | 61.89 | 15.02 | 61.89 | 20.70 | 72.58 | 16.89 | 72.10 | 23.81 | 64.93 | 18.02 | 61.30 | 22.40 |
| Dyspnea-pre | 0.67 | 1.12 | 0.00 | 0.75 | 0.37 | 0.76 | 0.00 | 0.50 | 0.74 | 1.09 | 0.00 | 1.00 |
| Fatigue-pre | 1.33 | 1.37 | 1.00 | 2.00 | 0.50 | 0.98 | 0.00 | 0.50 | 0.96 | 1.24 | 0.50 | 1.50 |
| Dyspnea-post | 2.39 | 1.37 | 2.00 | 1.50 | 2.09 | 1.50 | 2.00 | 2.00 | 2.39 | 1.26 | 3.00 | 1.50 |
| Fatigue-post | 3.07 | 2.13 | 3.00 | 2.00 | 2.37 | 1.39 | 2.00 | 2.50 | 2.22 | 1.46 | 2.00 | 2.50 |

**6**

**RhinAsthma Quest (0-100%)**

| Total score | 25.98 | 18.33 | 19.79 | 27.01 | 16.10 | 15.07 | 10.86 | 9.35 | 18.46 | 15.75 | 13.14 | 15.34 |
| --- | --- | --- | --- | --- | --- | --- | --- | --- | --- | --- | --- | --- |
| Limitation in daily life | 26.38 | 20.72 | 24.44 | 22.22 | 17.58 | 20.28 | 11.11 | 16.67 | 14.40 | 13.35 | 11.11 | 16.67 |
| Respiratory problems | 25.43 | 18.13 | 24.24 | 21.21 | 12.91 | 11.64 | 9.09 | 12.12 | 14.62 | 12.94 | 9.09 | 13.64 |
| Rhino-conjunctivitis score | 23.60 | 18.69 | 19.05 | 28.57 | 11.59 | 15.25 | 4.76 | 11.90 | 28.16 | 25.44 | 23.81 | 38.10 |
| Treatment & medication problems | 26.96 | 31.69 | 13.33 | 43.33 | 17.39 | 26.65 | 6.67 | 23.33 | 11.59 | 23.80 | 0.00 | 10.00 |
| Impairment in sensory perceptions | 27.54 | 19.37 | 25.00 | 20.83 | 21.01 | 18.10 | 16.67 | 25.00 | 23.55 | 21.85 | 16.67 | 33.33 |
| **Symptom Scores** | | | | | | | | | | | | |
| Asthma Control Test | 22.17 | 3.04 | 23.00 | 3.50 | 23.43 | 2.11 | 24.00 | 2.00 | 23.48 | 2.37 | 24.00 | 1.50 |
| VAS Allergy (0-100%) | 75.87 | 14.44 | 78.00 | 11.00 | 85.09 | 14.38 | 87.00 | 17.00 | 79.22 | 19.62 | 85.00 | 25.00 |
| VAS Health Status (0-100%) | 78.30 | 17.47 | 79.00 | 21.50 | 88.39 | 8.25 | 89.00 | 13.00 | 76.04 | 20.68 | 81.00 | 18.50 |

Table 2: Exercise-Group

day 0 day 10 day 60

Exercise-Group Mean SD Median IQR Mean SD Median IQR Mean SD Median IQR

**Physiology**

| Pulse (bpm) | 70.99 | 12.57 | 70.38 | 23.00 | 69.61 | 6.98 | 69.00 | 10.25 | 66.89 | 9.39 | 67.50 | 9.00 |
| --- | --- | --- | --- | --- | --- | --- | --- | --- | --- | --- | --- | --- |
| BP-Systole (mmHg) | 125.28 | 14.46 | 123.50 | 14.50 | 126.44 | 13.60 | 124.00 | 22.25 | 125.72 | 12.08 | 129.50 | 18.25 |
| BP-Diastole (mmHg) | 76.44 | 9.97 | 78.00 | 15.00 | 77.83 | 8.41 | 76.00 | 14.50 | 79.33 | 7.75 | 79.50 | 8.50 |
| FeNO oral (ppb) | 40.78 | 23.03 | 38.00 | 39.00 | 32.33 | 26.96 | 28.00 | 24.00 | 40.56 | 45.02 | 28.50 | 39.00 |
| FeNO nasal (ppb) | 465.33 | 178.07 | 451.00 | 146.00 | 396.44 | 180.80 | 375.50 | 245.25 | 383.67 | 184.37 | 394.50 | 204.25 |
| Nasal eosinophilic count (%) | 6.72 | 13.43 | 1.95 | 5.40 | 2.63 | 3.33 | 1.31 | 2.18 | 7.59 | 15.02 | 1.65 | 6.53 |
| Mucociliary Clearance (min) | 11.13 | 5.44 | 15.00 | 7.56 | 8.59 | 5.54 | 7.21 | 10.67 | 11.51 | 5.39 | 15.00 | 7.88 |

**Spirometry (%)**

| FVC | 108.17 | 17.66 | 108.00 | 12.75 | 108.33 | 15.88 | 108.00 | 10.75 | 111.50 | 13.46 | 110.00 | 10.25 |
| --- | --- | --- | --- | --- | --- | --- | --- | --- | --- | --- | --- | --- |
| FEV1 | 96.28 | 20.98 | 98.50 | 21.25 | 98.44 | 18.92 | 97.50 | 24.00 | 98.28 | 16.58 | 97.00 | 28.75 |
| FEV1/FVC | 93.39 | 11.81 | 95.00 | 17.00 | 94.83 | 10.79 | 98.00 | 17.00 | 92.39 | 10.75 | 94.00 | 18.25 |
| PEF | 99.50 | 26.37 | 102.50 | 35.00 | 99.33 | 22.09 | 100.00 | 25.00 | 103.17 | 20.69 | 99.00 | 26.75 |
| MEF25-75% | 73.33 | 31.74 | 71.50 | 33.50 | 78.61 | 30.67 | 75.50 | 35.75 | 73.28 | 29.91 | 71.00 | 32.75 |

**Differential Blood Count**

| WBC (103µl) | 7.41 | 1.81 | 7.20 | 2.25 | 6.42 | 1.30 | 6.16 | 1.48 | 7.01 | 1.12 | 6.92 | 1.22 |
| --- | --- | --- | --- | --- | --- | --- | --- | --- | --- | --- | --- | --- |
| RBC (106µl) | 4.82 | 0.37 | 4.79 | 0.41 | 4.79 | 0.39 | 4.79 | 0.34 | 4.79 | 0.36 | 4.68 | 0.44 |
| Blood neutrophil count (%) | 59.81 | 8.52 | 59.96 | 12.95 | 57.64 | 8.81 | 58.75 | 13.35 | 57.37 | 9.04 | 59.10 | 12.98 |
| Blood eosinophil count (%) | 3.85 | 2.95 | 3.35 | 1.92 | 4.28 | 4.85 | 2.90 | 3.35 | 4.18 | 3.90 | 2.95 | 2.50 |
| **Minute Walk Test** |  |  |  |  |  |  |  |  |  |  |  |  |
| Distance (%) | 104.35 | 8.17 | 105.99 | 10.97 | 106.13 | 6.59 | 105.75 | 11.25 | 105.69 | 7.72 | 106.80 | 11.78 |
| Peak respiratory frequency | 36.66 | 10.00 | 36.35 | 11.40 | 33.16 | 8.58 | 35.10 | 6.67 | 30.71 | 12.73 | 35.90 | 10.50 |
| Peak minute ventilation | 52.67 | 14.27 | 50.55 | 17.52 | 55.46 | 12.52 | 54.30 | 19.97 | 53.46 | 11.11 | 53.55 | 19.05 |
| Dyspnea-pre | 0.78 | 0.93 | 0.50 | 1.00 | 0.50 | 0.75 | 0.00 | 0.50 | 0.67 | 1.00 | 0.25 | 0.88 |
| Fatigue-pre | 0.97 | 1.02 | 0.75 | 1.75 | 0.64 | 1.44 | 0.00 | 0.50 | 0.61 | 1.08 | 0.00 | 0.88 |
| Dyspnea-post | 2.11 | 1.22 | 2.00 | 2.00 | 1.72 | 1.10 | 2.00 | 2.38 | 1.50 | 1.22 | 1.50 | 1.50 |
| Fatigue-post | 2.22 | 1.10 | 3.00 | 2.00 | 1.97 | 1.05 | 2.00 | 2.00 | 1.78 | 1.34 | 2.00 | 1.00 |

**6**

**RhinAsthma Quest (0-100%)**

| Total score | 19.09 | 12.20 | 15.87 | 20.18 | 14.80 | 10.97 | 11.27 | 13.06 | 14.20 | 12.70 | 7.75 | 10.66 |
| --- | --- | --- | --- | --- | --- | --- | --- | --- | --- | --- | --- | --- |
| Limitation in daily life | 15.80 | 12.48 | 11.11 | 23.89 | 12.72 | 11.68 | 10.00 | 18.89 | 11.98 | 12.10 | 8.89 | 22.78 |
| Respiratory problems | 20.37 | 14.24 | 16.67 | 14.39 | 16.16 | 14.55 | 10.61 | 18.18 | 15.66 | 13.60 | 12.12 | 14.39 |
| Rhino-conjunctivitis score | 21.69 | 15.77 | 21.43 | 26.19 | 11.90 | 11.11 | 9.52 | 13.10 | 15.61 | 16.15 | 9.52 | 16.67 |
| Treatment & medication problems | 14.44 | 14.86 | 10.00 | 18.33 | 11.48 | 16.93 | 6.67 | 13.33 | 9.26 | 16.19 | 0.00 | 13.33 |

Table 3: Control-Group

day 0 day 10 day 60

Control-Group Mean SD Median IQR Mean SD Median IQR Mean SD Median IQR

**Physiology**

| Pulse (bpm) | 68.14 | 8.81 | 67.00 | 10.75 | 69.18 | 7.92 | 69.50 | 9.00 | 68.73 | 8.58 | 69.50 | 8.75 |
| --- | --- | --- | --- | --- | --- | --- | --- | --- | --- | --- | --- | --- |
| BP-Systole (mmHg) | 118.45 | 12.35 | 117.00 | 20.00 | 116.32 | 9.74 | 116.00 | 14.00 | 115.45 | 8.85 | 116.00 | 12.50 |
| BP-Diastole (mmHg) | 72.95 | 10.19 | 70.00 | 17.75 | 70.73 | 7.72 | 69.50 | 9.50 | 70.77 | 8.56 | 69.00 | 8.75 |
| FeNO oral (ppb) | 38.91 | 22.30 | 36.00 | 22.50 | 38.45 | 26.55 | 32.50 | 26.25 | 52.05 | 49.94 | 39.00 | 21.75 |
| FeNO nasal (ppb) | 430.64 | 187.93 | 421.00 | 207.00 | 437.55 | 202.33 | 390.00 | 131.00 | 482.59 | 167.78 | 475.00 | 283.25 |
| Nasal eosinophilic count (%) | 5.70 | 14.43 | 1.16 | 1.56 | 3.71 | 4.77 | 1.53 | 3.94 | 8.21 | 10.84 | 3.21 | 7.22 |
| Mucociliary Clearance (min) | 12.43 | 5.67 | 13.62 | 6.81 | 11.20 | 5.10 | 11.75 | 7.19 | 11.66 | 4.59 | 15.00 | 7.07 |

**Spirometry (%)**

| FVC | 110.32 | 16.08 | 109.00 | 24.50 | 110.27 | 13.32 | 113.00 | 20.50 | 109.14 | 13.86 | 113.50 | 24.25 |
| --- | --- | --- | --- | --- | --- | --- | --- | --- | --- | --- | --- | --- |
| FEV1 | 103.73 | 13.21 | 99.50 | 18.50 | 104.27 | 11.42 | 104.50 | 19.50 | 102.91 | 11.62 | 103.00 | 18.75 |
| FEV1/FVC | 97.73 | 8.17 | 99.50 | 9.75 | 98.55 | 8.25 | 98.50 | 10.25 | 98.55 | 8.57 | 98.00 | 12.25 |
| PEF | 98.95 | 15.12 | 97.00 | 24.75 | 105.86 | 14.94 | 104.50 | 25.50 | 103.64 | 15.56 | 97.00 | 25.25 |
| MEF25-75% | 87.18 | 21.81 | 88.50 | 35.00 | 87.68 | 23.24 | 87.50 | 35.00 | 86.27 | 22.71 | 81.50 | 36.25 |

**Differential Blood Count**

| WBC (103µl) | 5.48 | 1.51 | 5.38 | 2.15 | 6.10 | 1.41 | 6.24 | 1.70 | 5.63 | 1.46 | 5.89 | 1.66 |
| --- | --- | --- | --- | --- | --- | --- | --- | --- | --- | --- | --- | --- |
| RBC (106µl) | 4.97 | 0.85 | 4.75 | 0.49 | 4.75 | 0.38 | 4.62 | 0.45 | 4.73 | 0.36 | 4.73 | 0.42 |
| Blood neutrophil count (%) | 55.55 | 8.03 | 56.00 | 10.38 | 56.71 | 7.58 | 56.90 | 9.50 | 56.53 | 7.28 | 56.70 | 11.02 |
| Blood eosinophil count (%) | 3.37 | 1.80 | 3.15 | 2.40 | 3.38 | 2.05 | 3.10 | 2.90 | 3.85 | 2.21 | 3.30 | 2.67 |
| **Minute Walk Test** |  |  |  |  |  |  |  |  |  |  |  |  |
| Distance (%) | 103.36 | 10.49 | 102.59 | 15.75 | 108.79 | 9.51 | 106.64 | 14.41 | 107.60 | 8.74 | 107.53 | 12.96 |
| Peak respiratory frequency | 30.78 | 4.08 | 31.10 | 4.50 | 36.55 | 8.11 | 34.10 | 10.52 | 35.31 | 7.09 | 35.70 | 7.45 |
| Peak minute ventilation | 50.64 | 16.06 | 47.35 | 14.85 | 56.30 | 16.02 | 55.80 | 17.73 | 54.97 | 17.82 | 55.05 | 26.07 |
| Dyspnea-pre | 0.50 | 0.79 | 0.00 | 1.00 | 0.55 | 0.90 | 0.00 | 0.88 | 0.36 | 0.76 | 0.00 | 0.50 |
| Fatigue-pre | 0.73 | 0.84 | 0.50 | 1.00 | 0.57 | 0.78 | 0.25 | 1.00 | 1.00 | 1.67 | 0.50 | 1.00 |
| Dyspnea-post | 1.73 | 1.07 | 2.00 | 1.00 | 1.95 | 1.67 | 2.00 | 2.38 | 1.55 | 1.66 | 1.00 | 1.50 |
| Fatigue-post | 1.61 | 1.22 | 2.00 | 1.38 | 2.07 | 1.91 | 2.00 | 2.38 | 1.93 | 1.89 | 2.00 | 1.38 |

**6**

**RhinAsthma Quest (0-100%)**

| Total score | 20.28 | 14.04 | 19.25 | 11.34 | 15.45 | 13.55 | 11.89 | 15.10 | 22.23 | 18.46 | 13.94 | 26.98 |
| --- | --- | --- | --- | --- | --- | --- | --- | --- | --- | --- | --- | --- |
| Limitation in daily life | 18.99 | 18.74 | 11.11 | 27.78 | 13.84 | 16.83 | 7.78 | 15.00 | 17.98 | 18.21 | 8.89 | 22.22 |
| Respiratory problems | 17.91 | 16.86 | 12.12 | 16.67 | 13.50 | 15.56 | 6.06 | 18.18 | 17.36 | 16.39 | 13.64 | 22.73 |
| Rhino-conjunctivitis score | 20.78 | 18.50 | 14.29 | 27.38 | 21.43 | 18.34 | 16.67 | 27.38 | 30.74 | 26.32 | 26.19 | 32.14 |
| Treatment & medication problems | 16.06 | 18.01 | 6.67 | 33.33 | 10.30 | 16.93 | 0.00 | 20.00 | 18.18 | 22.18 | 10.00 | 26.67 |
