# Supplementary material for: Winter Exercise and Speleotherapy for Allergy and Asthma: A Randomized Controlled Clinical Trial
Source: J Clin Med. 2020 Oct 15;9(10):3311. doi: 10.3390/jcm9103311 (PMC7602599; doi:10.3390/jcm9103311)
Supplement: Supplementary file 1 [file jcm-09-03311-s001.zip › Supplemental Sample Size Simulation.docx]

Table 1: Sample size simulation Exercise vs. Control group

Power

| Number | ANOVA | nparLD |
| --- | --- | --- |
| 10 | 0.31 | 0.36 |
| 20 | 0.64 | 0.61 |
| 30 | 0.84 | 0.78 |
| 40 | 0.95 | 0.89 |
| 50 | 0.97 | 0.94 |
| 60 | 0.99 | 0.96 |
| 70 | 1.00 | 0.99 |
| 80 | 1.00 | 1.00 |
| 90 | 1.00 | 1.00 |
| 100 | 1.00 | 1.00 |

Table 2: Sample size simulation Exercise vs. Speleotherapy group

Power

| Number | ANOVA | nparLD |
| --- | --- | --- |
| 10 | 0.25 | 0.26 |
| 20 | 0.41 | 0.49 |
| 30 | 0.54 | 0.71 |
| 40 | 0.64 | 0.82 |
| 50 | 0.74 | 0.89 |
| 60 | 0.82 | 0.94 |
| 70 | 0.87 | 0.98 |
| 80 | 0.91 | 0.99 |
| 90 | 0.94 | 0.99 |
| 100 | 0.96 | 1.00 |

1
